# ANGPTL4 Regulates Lung Adenocarcinoma Pyroptosis and Apoptosis via NLRP3\ASC\Caspase 8 Signaling Pathway to Promote Resistance to Gefitinib

**DOI:** 10.1155/2022/3623570

**Published:** 2022-11-25

**Authors:** Yue Fang, Xuan Li, Hao Cheng, Lu Zhang, Jiqing Hao

**Affiliations:** ^1^The First Affiliated Hospital of Anhui Medical University, Hefei 230022, Anhui, China; ^2^Hefei Cancer Hospital, Chinese Academy of Sciences, Hefei 230022, Anhui, China

## Abstract

**Background:**

Prior research has identified ANGPTL4 as a key player in the control of the body's lipid and glucose metabolism and a contributor to the onset of numerous cardiovascular conditions. Recently, it has been shown that ANGPTL4 also plays a critical role in tumor growth and progression. Nowadays, the number of EGFR-TKI resistant patients is increasing, and it is important to investigate the role of ANGPTL4 in regulating gefitinib resistance in PC9/GR non-small-cell lung cancer (NSCLC).

**Methods:**

The expression of ANGPTL4 in A549, PC9, H1975, BEAS-2B and PC9/GR cells was verified by Western blot and qRT-PCR assays, and the effect of gefitinib on the proliferative ability of each cell was probed by CCK-8 assay. By using shRNA to inhibit ANGPTL4 expression in cells, the effect of ANGPTL4 on cell migratory ability was examined and the effect of ANGPTL4 on cellular gefitinib sensitivity was confirmed using the CCK-8 assay and the edu proliferation test. Mouse transplantation tumors were constructed, and the effect of ANGPTL4 on cellular gefitinib sensitivity was investigated in vivo by flow cytometry, Tunel staining assay, immunohistochemical staining, and ROS fluorescence staining assay. ANGPTL4 expression in homoRNA overexpression cells was constructed, and the changes in the expression levels of ASC\NLRP3\Caspase 8 pathway and focal and apoptotic proteins were investigated in vitro, in vivo, afterknockdown and overexpression of ANGPTL4 expression by Westen blot assay.

**Results:**

ANGPTL4 was highly expressed in PC9/GR cells. Interfering with ANGPTL4 expression resulted in decreased proliferation and migration ability, decreased resistance to gefitinib, and increased scorching and apoptosis in PC9/GR cells. Interfering with ANGPTL4 expression in PC9/GR cells was shown to promote sensitivity to gefitinib and to mediate the NLRP3/ASC/Caspase 8 pathway to induce cell scorching and apoptosis.

**Conclusions:**

ANGPTL4 promotes gefitinib resistance in PC9/GR cells by regulating the NLRP3/ASC/Caspase 8 pathway to inhibit scorch death. ANGPTL4 may be an effective new target for inhibiting EGFR-TKI resistance in lung adenocarcinoma cells.

## 1. Background

For many years, lung cancer has been one of the most common malignant tumours worldwide, accounting for approximately 21% of the incidence and 27% of the mortality of all cancers. Approximately 49 of every 100,000 people in China die of lung cancer [1, 2]. Non-small-cell lung cancer accounts for approximately 85% of the total lung cancer cases and is associated with a five-year survival rate of only 15% in China [3]. Non-small-cell lung cancer shows heterogeneity. Mutations in multiple genes, including epidermal growth factor receptor (*EGFR*), anaplastic lymphoma kinase (*ALK*), *ROS1*, and *KRAS*, can promote the progression of this cancer. EGFR mutation is the most common gene mutation among patients with non-small-cell lung cancer and occurs in 50%–60% of Asian patients; most patients with these mutations have a low survival rate [4]. Treatment with EGFR-tyrosine kinase inhibitors (TKIs) is associated with progression-free survival (PFS) of 10–14 months in patients with EGFR-positive mutations [5].

Adenosine triphosphate cannot bind to the tyrosine kinase region of the intracellular domain of EGFR when used in combination with the first-generation EGFR-TKI medication gefitinib. This prevents the receptor from being phosphorylated [6]. Meta-analyses have shown that using gefitinib as a first-line treatment can improve the PFS of these patients, whereas combined chemotherapy and antiangiogenesis therapy can improve prognosis [7–9]. However, some patients show acquired drug resistance after 9–14 months of treatment, which affects the overall survival (OS) rate of patients [10].

Angiopoietin-like 4 (ANGPTL4) is widely present in the liver and adipose tissue and can regulate the activity of lipoprotein lipase by regulating the nutritional status of the body to regulate lipid metabolism [11]. Because of its important role in lipid metabolism, ANGPTL4 was once considered a regulator of lipid metabolism, but, in recent years, studies have found that ANGPTL4 is closely related to the proliferation and metastasis of a variety of malignant tumours. For instance, Zhu et al. [12] discovered that ANGPTL4 is substantially expressed in non-small-cell lung cancer tissues and is linked to the advancement of the malignancy and a poor prognosis. Furthermore, Gong et al. [13] found that ANGPTL4 promotes the occurrence of brain metastasis of breast cancer through the TGF-*β*2/ANGPTL4 axis. In addition, some researchers believe ANGPTL4 is closely related to the prognosis of malignant tumours, such as thyroid, cervical, and pancreatic cancer [14, 15]. Zhou et al. found high expression of ANGPTL4 in ovarian cancer cells and a positive correlation with secondary resistance to carboplatin [16], suggesting that ANGPTL4 may be associated with secondary resistance in the treatment of tumour patients. However, to the best of our knowledge, there have been no studies on the mechanism of acquired EGFR-TKI resistance in relation to ANGPTL4 in lung adenocarcinoma cells, and it remains unknown whether ANGPTL4 is involved in EGFR-TKI resistance.

By examining the characteristics of gefitinib-resistant PC9/GR cells in nonsmall cell lung cancer cells, we investigated the function and mechanism of ANGPTL4 in the process of acquiring drug resistance in lung adenocarcinoma in the current study. The results confirm that ANGPTL4 promotes the development of resistance to EGFR-TKIs in lung adenocarcinoma cells and that ANGPTL4 may be a potential target for overcoming resistance to EGFR-TKIs.

## 2. Method

### 2.1. Cells and Culture Conditions

The PC9, A549, H1945 (lung adenocarcinoma cells, Beijing Cell Bank, Beijing, China), BEAS-2B (bronchial epithelial cells, Beijing Cell Bank), and PC9/GR (gefitinib-resistant lung adenocarcinoma cells, Cell Center of Central South University, Changsha, China) cells were cultured in Dulbecco's modified Eagle medium supplemented with 10% foetal bovine serum and 1% penicillin-streptomycin solution. The PC9/GR cell culture medium was supplemented with 1 *μ*mol/L gefitinib to maintain cell resistance. Cells were cultured at 37°C with 5% CO_2_ in cell incubators. The medium was changed every 24 h, and a passage was conducted every 48 h. Cells were cryopreserved or resuscitated as required.

### 2.2. Short Hairpin (sh)RNA Transfection

ANGPTL4 expression was interfered with by transfecting shRNA into cells. Three shRNAs were purchased from Jikai Gene (Shanghai, China). The shRNA sequences were as follows: ANGPTL4-shRNA1, CCACAAGCACCTAGACCAT;ANGPTL4-shRNA2, ACAGCAGGATCCAGCAACT;ANGPTL4-shRNA3, ATCTTGGA AACTTGTGGACA. The control group was transfected with an empty vector (NC-shRNA)—the sequence was TTCTCCGAACGTGTCACGT. The PC9/GR cells were evenly spread into a six-well plate, with approximately 1 × 10^5^ cells/well, and incubated at 37°C for 16–24 h. When the cell fusion degree reached 70–80%, we configured the lip3000-shRNA liposome complex by allowing the Lipofectamine 3000 reagent to react with shRNA at 25°C for 20 min and added this to each well. The transfected cells were cultured for 12 h, and the medium was changed. After 48 h, fluorescence was observed under an inverted fluorescence microscope (Thermo Fisher Scientific, Shanghai, China). After 48–72 h of transfection, the efficiency was detected based on mRNA and protein expression using quantitative reverse transcription-polymerase chain reaction (qRT-PCR) and western blotting.

HomoRNA was used to increase the expression level of ANGPTL4. The former, the sequence of which was TCCAGGTTGGGGAGAGGCAGAGTGGACTAT, was purchased from Wanlei Biotechnology (Shenyang, China). The Lipofectamine 3000-homoRNA liposome complex was configured as described in the previous section to transfect PC9/GR cells, and the transfection efficiency was evaluated using western blotting.

### 2.3. qRT-PCR

Cellular mRNA or mouse tumor total RNA was extracted using the RNAeasy Animal RNA Extraction Kit (Beyotime, Shanghai, China). Then, we used the PrimeScript One-step RT-PCR Kit (Takara, Kyoto, Japan) to reverse transcribe mRNA into cDNA, which was subjected to PCR using LightCycler96 PCR (Roche, Basel, Switzerland) and the TB Green Premix Ex Taq II Kit (Takara). Using glyceraldehyde 3-phosphate dehydrogenase as an internal reference, the gene expression level was calculated according to formula 2^−ΔΔCT^. The names and sequences of the primers used in the experiment are shown in Table 1.

### 2.4. Western Blot

We gently rinsed the cells in the six-well plate with phosphate-buffered saline (PBS) and added 200–300 *μ*L of preconfigured radioimmunoprecipitation assay buffer and phenylmethylsulfonyl fluoride (99 : 1) cell lysate to each well. After completing cell lysis, the sample was centrifuged at 4°C (12,000 × g, 10 min). Then, we added 5× loading buffer (1/5^th^ of the supernatant volume) and placed the tube containing this mixture in a 100°C water bath for 10 min. After 10% SDS-PAGE, the bands were transferred to a polyvinylidene fluoride membrane and incubated with specific antibodies against ANGPTL4 (1 : 1000), NOD-like receptor thermal protein domain associated protein 3 (NLRP3, 1 : 2000), B-cell lymphoma 2 (BCL-2, 1 : 2000), apoptosis-associatedspeck-like protein containing CARD (ASC, 1 : 1000), cellular FLICE-like inhibitory protein (cFLIPL, 1 : 2000), caspase-8 (1 : 1500), cleaved caspase-8 (1 : 1500), and *β*-actin (1 : 2000) (Abcam, Cambridge, MA, USA). The next day, after incubation with IgG (H&L)-HRP antibody (1 : 5000), the bands were visualised on an integrated chemiluminescence imager helped by an enhanced chemiluminescence exposure solution.

### 2.5. Cell Viability Assay

Cells were spread evenly on a 96-well plate (3 × 10^4^ cells/well). After culturing for 24 h, the cells were switched to a 5% serum medium containing different concentrations of gefitinib (0.01, 0.5, 1, 2, 4, 8, 16, 32, and 100 *μ*mol/L) and 10 *μ*L of CCK-8 solution was added to each well after 48 h. The absorbance was measured at 570 nm after incubation for 2 h. Cell survival rate was calculated as follows: cell survival rate (%) = ((administration group *X* − negative control group *X*)/(nonadministration group *X* − negative control group *X*)) × 100.

### 2.6. Transwell

A 12-well plate was used to construct a small chamber model; the cells were spread in the upper chamber (6 × 10^4^ cells/well) with 200 *μ*L/well medium, and 400 *μ*L/well medium was added to the lower chamber. After culturing for 24 h, we gently wiped away the remaining cells from the upper chamber and placed the lower chamber in a 4% paraformaldehyde solution for 30 min; after subjecting the cells to 0.4% crystal violet staining, we used a microscope to observe the cells, as well as to count them in three randomly selected fields/well.

### 2.7. Apoptosis Detection by Flow Cytometry

The stabilised PC9/GR cells were plated into a six-well plate (4 × 10^5^ cells/well), and the cells were cultured for 48 h in a medium containing different concentrations of gefitinib (0.5 and 8 *μ*mol/L). After digestion and centrifugation (500 × g, 5 min), the cell density was adjusted to 1 × 10^6^/mL, and the apoptosis level was measured using flow cytometry (FACScan, Shanghai, China) with an Annexin-VFITC/PI Apoptosis Kit (Univ, Shanghai, China).

### 2.8. EDU Cell Proliferation Assay

After adjusting the cell status, we transferred the cells in the logarithmic phase into a six-well plate (4 × 10^6^ cells/well). When cell confluence reached 70%, we added prewarmed EdU staining solution (a final concentration of 10 *μ*M/L) to each group of cells. After incubating for 3 h, we fixed them with 4% paraformaldehyde for 20 min at 25°C. Subsequently, we washed them twice with PBS, added 0.1 mL of 0.5% Triton *X*-100 (Thermo Fisher Scientific, Shanghai, China) in PBS to each well, incubated them at 25°C for 20 min, washed them twice, added Click-iT (Thermo Fisher Scientific) to the reaction solution, incubated them at 25°C for 30 min in the dark, washed the cells twice, and treated them with diluted Hoechst 33342 staining solution (1 : 2000; Thermo Fisher Scientific) for 15 min. After staining, the cells were washed twice again with PBS and photographed under a fluorescence microscope.

### 2.9. Nude Mouse Xenograft Model

Six-week-old BALB/c nude female mice, purchased from Beijing Vitalriver Experimental Animal Technology, were randomly divided into three groups (8 mice in each group, *n* = 24). PC-9/GR cells, Sh-NC PC-9/GR cells, and Sh-ANGPTL4 PC9/GR cells mixed with an equal number of Matrigel-9/GR cells (1 × 10^6^) were injected subcutaneously into the left side of the mouse to construct a nude xenograft mouse model (100 *μ*L each). All mice were fed in a specific pathogen-free animal room. When the tumor was palpable, the volume of the subcutaneously transplanted tumor was measured and recorded daily. As the tumour volume reached 50 mm^3^ (calculation for volume: long diameter × short diameter × short diameter/2), the ANGPTL4-shRNA components were divided into two groups: *A* and *B*. To assess the survival rate of nude mice, the control group, NC-shRNA group, and ANGPTL4-shRNA A group were orally administered 150 mg of gefitinib/day for treatment, and the ANGPTL4-shRNA B group was orally administered an equal volume of PBS. The mice were weighed and photographed every 3 days, and the body weight curve was drawn. After 21 days of oral administration, all mice were euthanised and the tumour tissues were excised and photographed; in addition, the long diameter, short diameter, and tumor volume were calculated, and the weight of the tumour was measured. All animal experiments were conducted according to the institutional guidelines of the Animal Care and Use Committee of the First Affiliated Hospital of Anhui University.

### 2.10. Immunohistochemistry

Slices of the tissue embedded in wax were sequentially placed in xylene I and II; absolute ethanol I and II; 95%, 85%, and 75% ethanol; and distilled water. Subsequently, they were soaked in PBS and wiped dry. Slices were incubated in 3% H_2_O_2_ for 15 min at 25°C and soaked in PBS for 5 min three times. They were incubated in 1% BSA at 25°C for 15 min and a mixture of antibodies (1 : 50) overnight in a humid environment. On the next day, we washed them twice with PBS (7 min/wash). The IgG (H&L)-HRP antibody (1 : 500) was added dropwise to the tissue, which was subsequently placed in a humid environment for 1 h. After the reaction, the cells were washed with PBS for 10 min. After washing, DAB staining solution and haematoxylin counterstaining were performed. The counterstained sections were dehydrated and sealed with neutral gum. The sections were observed under a microscope and photographed.

### 2.11. Detection of Apoptosis by Tunel Assay

After deparaffinisation of the tissue sections, 45 *μ*L of 0.1% Triton X-100 was added to them dropwise. The sections were then placed at 25°C for 7 min and washed with PBS. Subsequently, they were treated with 50 *μ*L of preconfigured terminal deoxynucleotidyl transferase-mediated reaction solution of dUTP-biotin nick end labelling (TUNEL) (enzyme solution: label solution = 1 : 9) for 1 h, washed with PBS, counterstained with 4′,6-diamidino-2-phenylindole, and incubated in the dark for 4 min. The residual reagent was washed away with PBS, and the fluorescent quencher was added dropwise to mount the slide. The sections were examined and photographed using an inverted fluorescence microscope.

### 2.12. ROS Fluorescence Staining

After the frozen portions had dried, the tissue was circled with a histochemical pen. 400 *μ*l of ROS staining solution (Servicebio G1045) was added dropwise to the circles and incubated for 30 min at 37°C in the dark. The slides were washed three times for 6 min each time by shaking in PBS on a decolorizing shaker.

The slides were slightly shaken and dried; then, DAPI staining solution was added dropwise in a circle and incubated at room temperature for 10 min in the dark. The slides were then soaked in PBS and washed 3 times with shaking on a decolorizing shaker for 5 min each time. The slices were slightly shaken and then sealed with an antifluorescence quenching sealer. Slices were observed under a fluorescence microscope, and images were acquired (emission wavelength 515–555 nm).

### 2.13. Statistics

All experiments in this study were repeated three times. GraphPad Prism software (version 7.0) (GraphPad Software, Beijing, China) was used to generate graphs, and SPSS 22.0 (IBM, Armonk, New York, NY, USA) was used for the statistical analysis of the data. The experimental data are expressed as mean ± standard deviation ($\bar{x}\pm s$). We used the *t*-test for comparisons between groups and a one-way analysis of variance for comparison among multiple groups. The threshold of statistical significance was set at *p* < 0.05.

## 3. Results

### 3.1. ANGPTL4 Is Highly Expressed in PC9 and PC9/GR Cells

**Figure 1:**
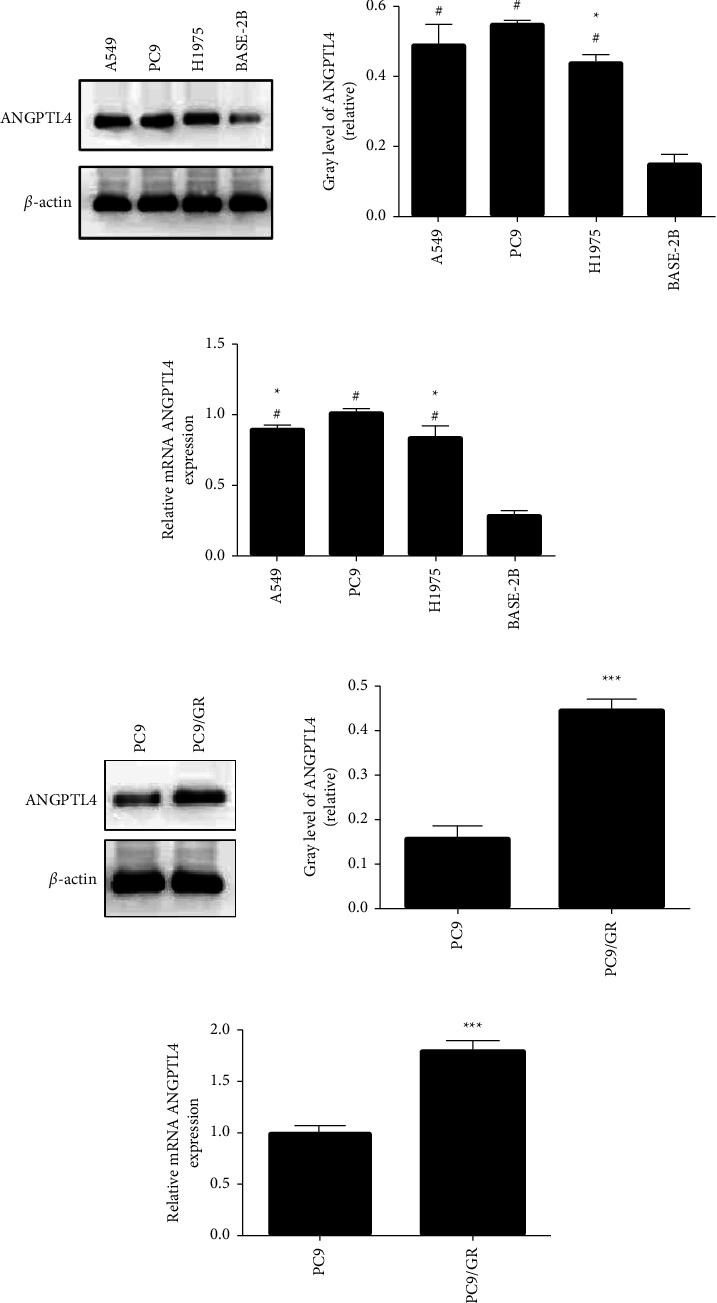
ANGPTL4 expression in A549, PC9, BEAS-2B, and PC9/GR cells ($\bar{x}\pm s$, *n* = 3). (a, b) ANGPTL4 protein and mRNA expression in A549, PC9, and BEAS-2B cells; (c) CCK-8 test revealed the IC_50_ in A549, PC9, and BEAS-2B cells; (c, d) ANGPTL4 protein and mRNA expression in PC9 and PC9/GR cells. ^#^*p* *<* 0.001 compared to the BEAS-2B group; ^*∗*^, ^*∗∗*^, and ^*∗∗∗*^*p* *<* 0.05, 0.01, and 0.001, respectively, compared to the PC9 cell group.

To explore ANGPTL4 expression in A549, PC9, H1975, and BEAS-2B cell lines, we performed qRT-PCR and western blotting. ANGPTL4 expression was very low in BEAS-2B cells but high in A549, PC9, and H1975 cells (*p* < 0.05); mRNA expression in PC9 cells was higher than that in A549 and H1975 cells (*p* < 0.05, Figures 1(a) and 1(b)). Therefore, the PC9 gefitinib-resistant cells and PC9 cells were cultured for subsequent experiments.

Furthermore, ANGPTL4 mRNA and protein expression in PC9 and PC9/GR cells was analysed, and it was found that ANGPTL4 expression in PC9/GR cells was significantly higher than that in PC9 cells (*p* *<* 0.05; Figures 1(c) and 1(d)).

### 3.2. ANGPTL4 Is Associated with Resistance to Gefitinib in Lung Adenocarcinoma Cells

**Figure 2:**
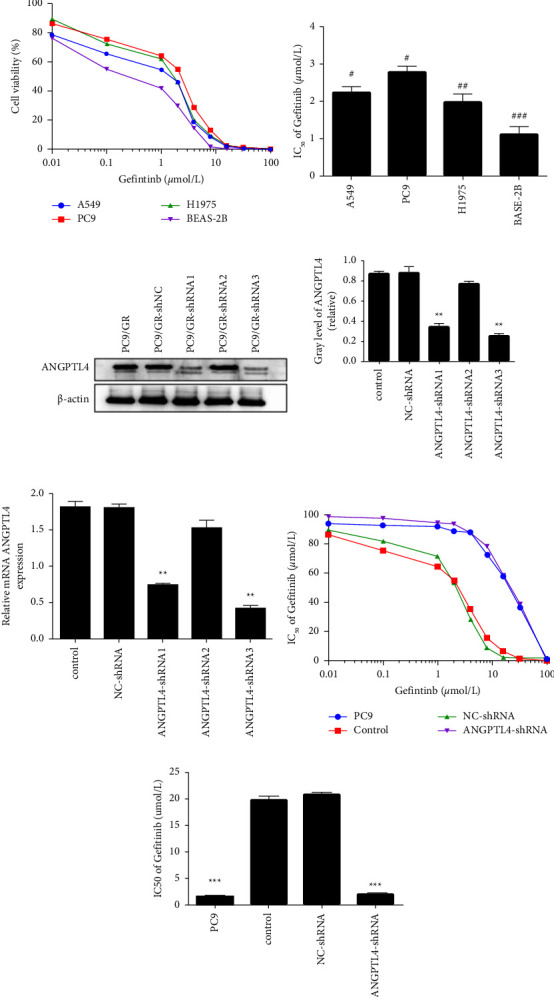
Knockdown with ANGPTL4 expression reduces the resistance of PC9/GR to gefitinib ($\bar{x}\pm s$, *n* = 3). (a, b) The CCK-8 test revealed the cell viability and IC_50_ of gefitinib in A549, PC9, H1975, and BEAS-2B cells. (c, d) ANGPTL4 protein and mRNA expression in PC9/GR cells after transfection with ANGPTL4-shRNA and NC-shRNA. (e, f) CCK-8 test revealed the cell viability and IC_50_ of gefitinib in PC9/GR cells after ANGPTL4 knockdown. Control: blank control group; NC-shRNA: PC9/GR cells transfected with NC-shRNA; G: PC9/GR cells + gefitinib; ANGPTL4-shRNA: PC9/GR cells transfected with ANGPTL4-shRNA; ANGPTL4-shRNA + G: PC9/GR cells transfected with ANGPTL4-shRNA + gefitinib. ^*∗*^, ^*∗∗*^, and ^*∗∗∗*^: *p* < 0.05, 0.01, and 0.001, respectively, compared to the control group; ^#^, ^##^ and ^###^: *p* < 0.05, 0.01, and 0.001, respectively, compared to the PC9 group.

The effect of different concentrations of gefitinib on the cell viability of A549, PC9, H1975, and BEAS-2B cells was investigated by CCK-8 assay. The IC50 of A549, PC9, H1975, and BEAS-2B cells was 2.44 ± 0.36, 2.93 ± 0.27, 2.23 ± 0.43 and 1.35 ± 0.51 *μ*mol/l, respectively, with the IC50 of PC9 cells being significantly higher than the other three groups (Figures 2(a) and 2(b), *p* < 0.05).

Then, we transfected PC9/GR cells with shRNA and performed western blotting and qRT-PCR to verify the knockdown effect. We found that both shRNA1 and shRNA3 had a stable knockdown effect (*p* < 0.01) and that the knockdown effect of shRNA3 was more evident than that of shRNA1 (Figures 2(c) and 2(d)).

In subsequent experiments, shRNA3 was used to knock down ANGPTL4. Cell viability studies on PC9, PC9/GR, NC-shRNA, and ANGPTL4-shRNA3 cells revealed the IC_50_ values of the PC9 group (2.597 ± 0.154 *μ*mol/L) and ANGPTL4-shRNA3 group (2.817 ± 0.245 *μ*mol/L) were lower than those of the PC9/GR group (20.73 ± 0.25 *μ*mol/L; both, *p* < 0.001) and NC-shRNA (20.71 ± 0.592 *μ*mol/L; *p* > 0.05; Figures 2(e) and 2(f)).

### 3.3. ANGPTL4 Correlates with the Level of Invasion and Apoptosis of PC9/GR Cells

**Figure 3:**
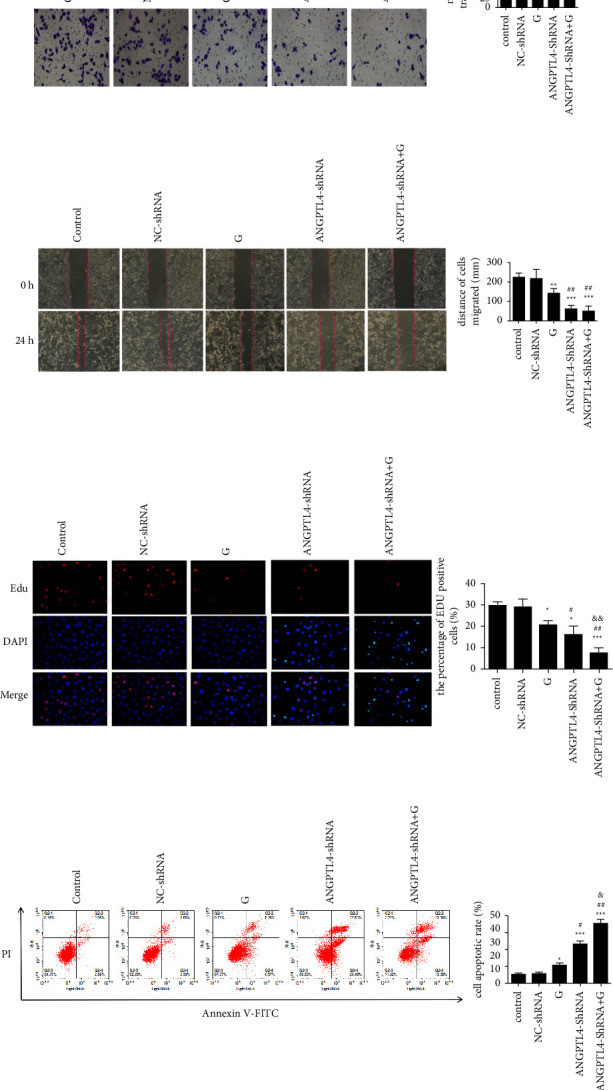
Knockdown with ANGPTL4 expression reduces the resistance of PC9/GR to gefitinib ($\bar{x}\pm s$, *n* = 3). (a) Transwell test revealed the correlation between ANGPTL4 knockdown and PC9/GR cell migration ability (×400). (b) Scratching experiments revealed a correlation between ANGPTL4 knockdown and the migration ability of PC9/GR cells (×200). (c) EdU proliferation test revealed the correlation between ANGPTL4 knockdown and PC9/GR cell proliferation (×400). (d) Flow cytometry showed the correlation between ANGPTL4 knockdown and PC9/GR cell apoptosis. Control: blank control group; NC-shRNA: PC9/GR cells transfected with NC-shRNA;*G*: PC9/GR cells + gefitinib; ANGPTL4-shRNA: PC9/GR cells transfected with ANGPTL4-shRNA; ANGPTL4-shRNA + G: PC9/GR cells transfected with ANGPTL4-shRNA + gefitinib. ^*∗*^, ^*∗∗*^, and ^*∗∗∗*^: *p* < 0.05, 0.01, and 0.001, respectively, compared to the control group; ^#^ and ^##^: *p* < 0.05 and 0.01, respectively, compared to the G group; ^&^, ^&&^, and ^&&&^: *p* < 0.05, 0.01, and 0.001, respectively, compared to the ANGPTL4-shRNA group.

The results of the Transwell assay and scratch assay showed (Figures 3(a) and 3(b)) that increasing gefitinib concentration could inhibit PC9/GR cell invasion and migration, but the effect was lower than interfering with ANGPTL4 expression, and knocking down ANGPTL4 while increasing gefitinib drug induction could further inhibit PC9/GR cell invasion and migration ability. The results of flow cytometry and Edu proliferation assays showed that the knockdown of ANGPTL4 expression inhibited PC9/GR cell proliferation and promoted apoptosis (Figures 3(c) and 3(d)), while knockdown of ANGPTL4 expression significantly inhibited PC9/GR cell proliferation and promoted apoptosis under gefitinib induction. The above experimental results showed that ANGPTL4 positively correlated with the invasive migration and proliferation viability of PC9/GR cells, while negatively correlated with the apoptosis level.

### 3.4. Interference with ANGPTL4 Expression In Vivo can Inhibit Tumour Progression

**Figure 4:**
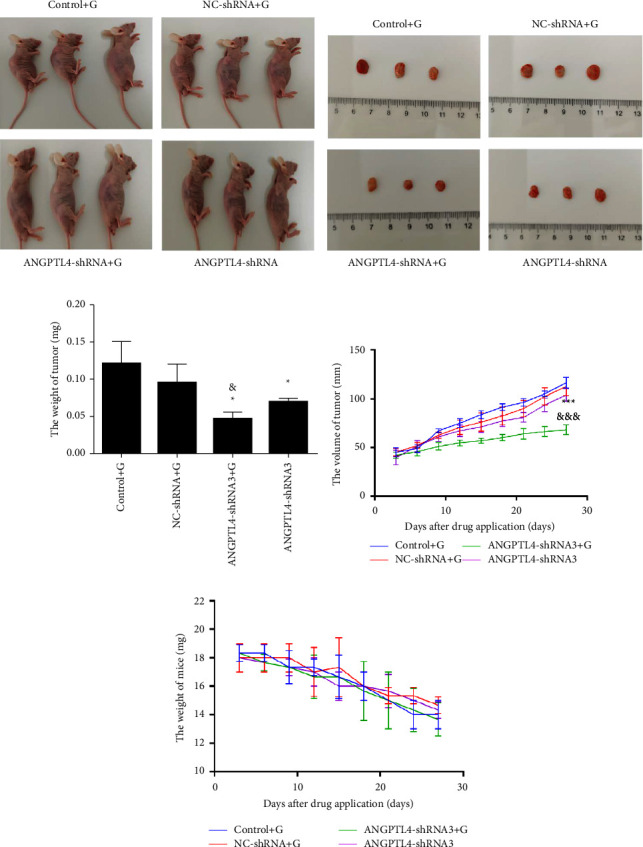
Knockdown with ANGPTL4 has a significant antitumour effect on the xenograft tumour model ($\bar{x}\pm s$, *n* = 3). (a) Nude mouse xenograft tumour model. (b) Xenograft tumour in each group. (c) Nude mouse weight change curve. Comparison of xenograft tumour weights between groups. (d) Volume growth curves of xenograft tumours in each group. (e) Nude mouse weight change curve. ^*∗*^and ^*∗∗∗*^: *p* < 0.05 and 0.001, respectively, compared to the control + G group; ^&^and ^&&&^: *p* < 0.05 and 0.001, respectively, compared to the ANGPTL4-shRNA3 group.

To further understand the effect of ANGPTL4 on the acquired resistance of gefitinib, we used PC9/GR and ANGPTL4-shRNA cells to construct a nude xenograft mouse model (Figures 4(a) and 4(b)). Under the same treatment with gefitinib, the growth rate and weight of the tumor in the ANGPTL4-shRNA + G group were lower than those in the control + G group (*p* < 0.05, Figures 4(d) and 4(e)). However, there was no significant difference in the body weights of the mice in each group (Figure 4(c)).

**Figure 5:**
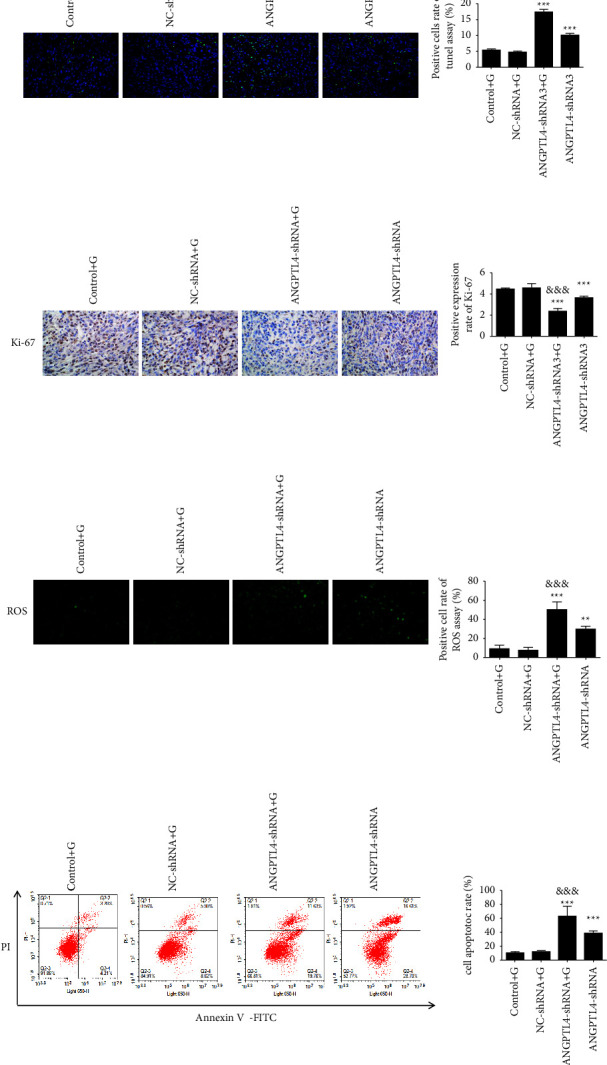
Effect of interfering ANGPTL4 on transplanted tumor tissue ($\bar{x}\pm s$, *n* = 3). (a) TUNEL assay was performed to detect the level of apoptosis in xenograft tissue (×400). (b) Ki-67 expression in the tumour tissue of each group. (c) ROS fluorescence staining was performed to detect the level of ROS in xenograft tissue (×400). (d) Flow cytometry showed the level of apoptosis in xenograft tissue. ^*∗*^and ^*∗∗∗*^: *p* < 0.05 and 0.001, respectively, compared to the control + G group; ^&^and ^&&&^: *p* < 0.05 and 0.001, respectively, compared to the ANGPTL4-shRNA3 group.

The TUNEL assay revealed that knockdown with ANGPTL4 expression increased the level of apoptosis (*p* < 0.001, Figure 5(a)). Furthermore, it showed that the apoptotic level of the ANGPTL4-shRNA group was higher than that of the control + G group (*p* < 0.001, Figure 5(d)). This suggests that ANGPTL4 is better than gefitinib for the regulation of apoptosis in vivo. Furthermore, immunohistochemical staining showed that Ki-67 levels in transplanted tumour tissue from the ANGPTL4-shRNA and ANGPTL4-shRNA + G groups were lower than those in the control + G group (both, *p* < 0.001), which suggests that ANGPTL4 promotes tumor proliferation in vivo (Figure 5(b)). Together, the inhibition of ANGPTL4 expression can inhibit tumor growth and proliferation.

The results of the ROS fluorescence staining experiment also showed that gefitinib treatment combined with reduction of ANGPTL4 increased the degree of ROS expression in PC9/GR cells.

### 3.5. ANGPTL4 Inhibits Pyroptosis and Apoptosis by Regulating the NLRP3\ASC\Caspase 8 Pathway

**Figure 6:**
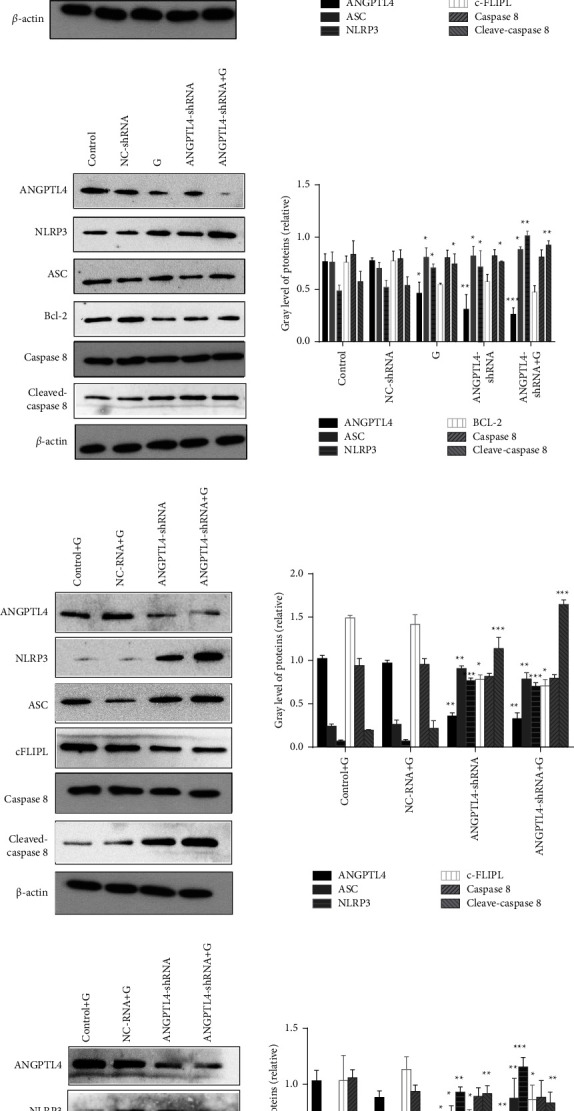
Changes in the expression of NLRP3\ASC\Caspase 8 pathway-related proteins after ANGPTL4 knockdown and overexpression in cells and transplanted tumour tissues ($\bar{x}\pm s$, *n* = 3). (a) NLRPS\ASC\Caspase 8 and pyroptosis-related protein expression after ANGPTL4 knockdown in PC9/GR cells. (b) Changes in the expression of NLRP3\ASC\Caspase 8 and apoptosis-related protein expression after ANGPTL4 knockdown in PC9/GR cells. (c) NLRP3\ASC\Caspase 8 and pyroptosis-related protein expression after ANGPTL4 knockdown in xenograft tumours. (d) NLRP3\ASC\Caspase 8 and apoptosis-related protein expression after ANGPTL4 knockdown in xenograft tumours. Control: PC9/GR cells; NC-shRNA: PC9/GR cells transfected with NC-shRNA;*G*: PC9/GR cells + gefitinib; ANGPTL4-shRNA: PC9/GR cells transfected with ANGPTL4-shRNA;ANGPTL4-shRNA + *G*: PC9/GR cells transfected with ANGPTL4-shRNA + gefitinib; Control + *G*: nude mice treated with gefitinib; NC-shRNA + *G*: nude mice treated with NC-shRNA cells + gefitinib; ANGPTL4-shRNA: nude mice treated with PC9/GR cells afterknockdown with ANGPTL4; ANGPTL4-shRNA + *G*: after knockdown with ANGPTL4 nude mice treated with PC9/GR cells + gefitinib. ^*∗*^, ^*∗∗*^ and ^*∗∗∗*^: *p* < 0.05,0.01 and 0.001, respectively, compared to the control or control + *G* group.

To explore the specific mechanism by which lung adenocarcinoma cells acquire gefitinib resistance, the expression levels of the pyroptosis-related proteins ACS, NLRP3, cFLIPL, and Caspase 8 in ANGPTL4 knockdown were detected by western blotting. In ANGPTL4-knockdown cells, NLRP3, ACS and cleaved-caspase 8 expression decreased and increased, respectively (*p* < 0.001, Figure 6(a)). In contrast, the expression levels of cFLIPL in ANGPTL4-knockdown cells were increased and decreased, respectively (*p* < 0.001, Figure 6(a)).

Since both in vivo and ex vivo research demonstrated that cells' levels of apoptosis rose after being knocked down with ANGPTL4, we also looked at the expression of proteins linked to apoptosis in the knockdown cells. After the knockdown of ANGPTL4, the expression of inhibitory proteins, such as Bcl-2, decreased significantly (*p* < 0.05, Figure 6(b)).

To confirm that ANGPTL4 can regulate the occurrence of pyroptosis and apoptosis through the NLRP3\ASC\Caspase 8 pathway, we used tissue proteins from the xenograft tumor to re-verify the results. Under the conditions of drug treatment and culture, in nude mice treated with ANGPTL4-shRNA cells, ASC, NLRP3, and cleaved-caspase 8 expression was higher than that in the control group and Bcl-2 and cFLIPL expression was lower than that in the control group (*p* < 0.05, Figures 6(c) and 6(d)). Collectively, ANGPTL4 inhibits lung adenocarcinoma cell pyroptosis and apoptosis by regulating the NLRP3\ASC\Caspase 8 pathway both in vivo and in vitro.

## 4. Discussion

More and more specialized medications have been created as targeted lung cancer therapy research has progressed. The most frequent location of mutation among the several targets is EGFR [4]. Currently, EGFR-TKIs have been developed to the fourth generation, while the first-generation drugs, represented by gefitinib, are still widely used in Asia and other regions for their high therapeutic effectiveness and low side effects [6, 17]. However, the issue regarding the secondary resistance to EGFR-TKIs in lung adenocarcinoma patients has still not been adequately addressed. In our previous study, we found high expression of ANGPTL4 in PC9/GR cells [18], suggesting that ANGPTL4 may be one of the potential targets for gefitinib resistance in lung adenocarcinoma cells. In this study, we further confirmed that high expression of ANGPTL4 promoted secondary resistance to PC9 gefitinib in lung adenocarcinoma cells through in vivo and ex vivo experiments and explored the specific mechanism, which helps to improve the understanding of the correlation between ANGPTL4 and resistance to EGFR-TKIs.

ANGPTL4 belongs to the ANGPTL superfamily and can regulate lipid metabolism, leading to coronary heart disease and many other cardiovascular diseases [19]. Previous studies have confirmed the high expression of ANGPTL4 in a variety of malignant tumor cells and tissues, such as pancreatic cancer [20], gastric cancer [21], cholangiocarcinoma cells [22], and breast cancer [13, 19]. In our study, by comparing the expression levels of ANGPTL4 in three different lung adenocarcinoma cells and human bronchial epithelial cells BEAS-2B, we found that the expression levels of ANGPTL4 were higher in all three lung adenocarcinoma cells than in BEAS-2B cells, demonstrating that ANGPTL4 expression was upregulated in lung adenocarcinoma cells. This finding is consistent with the study by Zhu et al. [12]. A gap in the literature exists regarding the relationship between ANGPTL4 and resistance to EGFR-TKIs, despite the fact that ANGPTL4 has been identified as a tumor-promoting factor based on the series of studies mentioned above that suggest it can be upregulated and contribute to the progression of a number of malignancies.

In our study, we found that the expression of ANGPTL4 was upregulated in PC9/GR cells, which was significantly higher than that in PC9 cells. And the resistance of cells to gefitinib was significantly decreased after knockdown with ANGPTL4 expression in PC9/GR cells. In addition, knockdown with ANGPTL4 led to a decrease in the proliferation, migration, and invasion ability of PC9/GR cells and an increase in the level of apoptosis and the expression level of ROS in vivo and vitro.

Consistent with our study, ANGPTL4 was also found to be negatively correlated with apoptosis and ROS levels in ovarian cancer cells in the study by Yang et al. [23].

Pyroptosis is the same as apoptosis, and both are a type of programmed cell death [24]. The onset of pyroptosis is often accompanied by chromatin condensation and DNA breakage, cell membrane pore formation, cell swelling, and membrane rupture, which in turn leads to the release of cellular contents and pro-inflammatory mediators [24].

The current study found that intracellular focal death is activated mainly through two pathways; one of them is the classic pathway that relies on GSDMD [25]. As a central target in the classical pathway, NLRP3 has been found to be aberrantly expressed in a variety of malignancies, such as ovarian cancer [26] and breast cancer [27]. However, to date, there are still no studies on the correlation between ANGPTL4 and pyroptosis.

In the present study, we observed that after discriminating between knockdown of ANGPTL4 in PC9/GR cells, expression of pyroptosis-related proteins NLRP3, ASC, and cleaved-caspase8 were subsequently upregulated and decreased, but the expression levels of the pyroptosis inhibitor protein cFLIPL were increasing and decreasing, respectively, in vivo and vitro. This suggests that ANGPTL4 regulates gefitinib resistance in lung adenocarcinoma cells through the NLRP3/ASC/Caspase8 pathway.

Additionally, according to certain research, pyroptotic cell death is frequently accompanied by both necrosis and apoptosis traits [28]. Moreover, our research discovered that levels of apoptosis in cells rose following ANGPTL4 knockdown. Fritsch et al. showed that caspase 8 is not only involved in the scorching of tumour cells but also plays an important role in the process of apoptosis [29]. In a study by Chi W et al., the expression levels of NLRP3, ASC, Caspase8, and apoptosis-related proteins were significantly increased in an acute diabetic mouse model [30]. Chen et al. further demonstrated that pannexin-1 promotes caspase-8 or caspase-9-dependent apoptosis by promoting the activation of NLRP3 inflammatory vesicles through the construction of GSDMD D88A knock-in mice [31]. Our study also found that the expression levels of NLRP3, ASC, and Cleaved-caspase 8 increased and decreased after knockdown and overexpression of ANGPTL4, respectively, while the expression levels of the apoptosis suppressor protein Bcl-2 were reversed suggesting that ANGPTL4 regulates apoptosis in PC9/GR cells through mediating the NLRP3/ASC/Caspase 8 pathway.

In summary, our research explored the regulatory ability and mechanism of ANGPTL4 in the resistance of PC9/GR cells to gefitinib through in vivo and in vitro experiments. ANGPTL4 inhibited pyroptosis and apoptosis by regulating the NLRP3/ASC/Caspase 8 pathway, leading to resistance to gefitinib in lung adenocarcinoma. ANGPTL4 may be an important regulator of EGFR-TKI resistance in patients with non-small-cell lung cancer. Targeting ANGPTL4 may inhibit the development of EGFR-TKI resistance, which may improve the survival rate of patients with non-small-cell lung cancer. However, our experiments had shortcomings. For example, due to the strong proliferation ability of PC9/GR cells, gefitinib treatment was added to the control group to improve the survival rate of nude mice, which may have led to the loss of the true blank control group in animal experiments.

## Figures and Tables

**Table 1:** qRT-PCR primer names and sequences.

Primers	Primer sequences (5′-3′)
ANGPTL4	F: GTCCACCGACCTCCCGTTA
	R: CCTCATGGTCTAGGTGCTTGT

GAPDH	F: GGAGCGAGATCCCTCCAAAAT
	R: GGCTGTTGTCATACTTCTCATGG

## Data Availability

The datasets used during the current study are available from the corresponding author upon request.
